# Brain-transportable soy dipeptide, Tyr-Pro, attenuates amyloid β peptide_25-35_-induced memory impairment in mice

**DOI:** 10.1038/s41538-020-0067-3

**Published:** 2020-05-01

**Authors:** Mitsuru Tanaka, Hayato Kiyohara, Atsuko Yoshino, Akihiro Nakano, Fuyuko Takata, Shinya Dohgu, Yasufumi Kataoka, Toshiro Matsui

**Affiliations:** 1 Laboratory of Food Analysis, Department of Bioscience and Biotechnology, Faculty of Agriculture, Graduate School of Kyushu University, 744 Motooka, Nishi-ku, Fukuoka, 819-0395 Japan; 2 Department of Pharmaceutical Care and Health Sciences, Faculty of Pharmaceutical Sciences, Fukuoka University, 8-19-1 Nanakuma, Jonan-ku, Fukuoka 814-0180 Japan

**Keywords:** Nutrition, Peptides

## Abstract

In this study, experiments on amyloid β peptide_25-35_-induced mice were performed to provide in vivo evidence on the potential of the blood–brain barrier transportable soy dipeptide, Tyr-Pro, in combating memory impairment. We demonstrated for the first time that oral administration of Tyr-Pro (100 mg/kg, twice a day) in mice for 16 days significantly improved impaired memory by spontaneous alternation and shortened step-through latency in amyloid β-induced mice.

## Introduction

In a recent study, we provided strong evidence that dipeptides possessing Pro, such as Gly-Pro and Tyr-Pro, can be transported across the blood–brain barrier (BBB) in an intact form into the parenchyma of peptide-perfused mouse brain^1^. Thus far, several animal reports have hinted at the memory-improving effect of peptides, such as Leu-His, which attenuates microglial activation and emotional disturbances^2^, Met-Lys-Pro^3^, and Trp-Tyr^4^, which prevent cognitive decline. In the report of administered Trp-[carboxyl-^14^C]Tyr^4^, radioactive substances were detected in mouse brain, while no evidence on accumulation of the intact dipeptide in the brain parenchyma was provided. In contrast, using our proposed phytic acid-aided MALDI-MS/MS imaging analysis^1,5^, we pointed out the first finding that the BBB-transportable Tyr-Pro from soybean hydrolysate^1^ subsequently accumulated in the hippocampus, cerebral cortex, hypothalamic area, striatum, and cerebellum of mouse brain. The accumulated regions mainly regulate memory^6^; therefore, in vivo experiments using memory-impaired mice will provide insight into the benefits of BBB-transportable Tyr-Pro against cognitive impairment. Amyloid β peptide (Aβ)_25-35_-induced mice were used for this study, since the Aβ_25-35_-induction was confirmed to cause the impairment of cognitive brain function^7,8^.

## Results and discussion

**Fig. 1:**
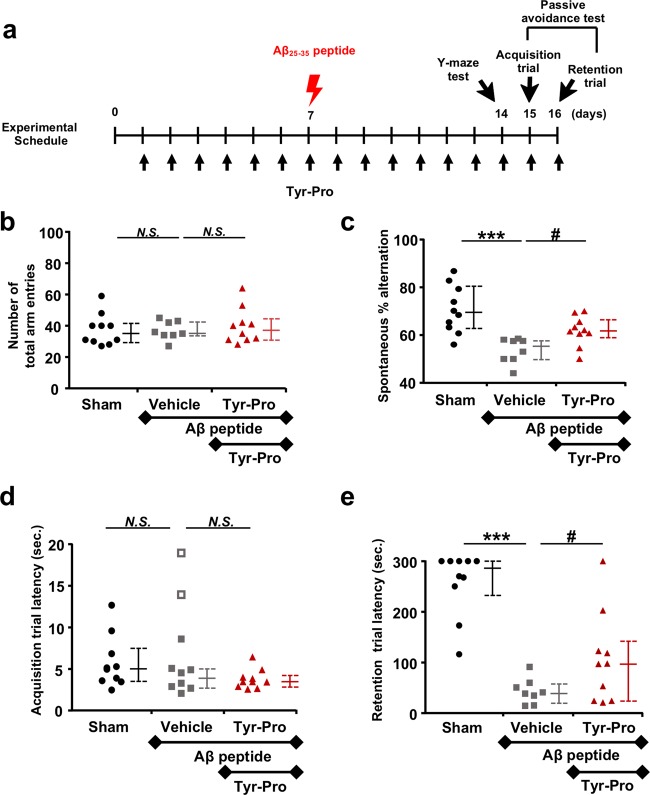
Effects of oral administration of Tyr-Pro (100 mg/kg, p.o.) for 16 days on Aβ_25-35_-induced impairment of working and long-term memories in Y-maze test and passive avoidance test, respectively. Tyr-Pro (100 mg/kg) was orally administered twice a day for 16 days, except for days of *i.c.v.* injection of Aβ_25–35_ peptide and the behavioural tests (Tyr-Pro administration once a day) (**a**). The mice received Aβ_25–35_ peptide injection (6 nmol/mouse, *i.c.v*.) on the 7th day and Tyr-Pro administration was performed after recovery from anaesthesia. The Y-maze test was started at 60 min after Tyr-Pro administration on the 14th day. The number of total arm entries (**b**) and percentage of spontaneous alternations (**c**) were evaluated. Acquisition trial on the 15th day (**d**) and the retention trial on the 16th day (**e**) were performed in the passive avoidance test. Both trials were started at 60 min after Tyr-Pro administration. Data for corresponding mice in sham (*n* = 10), vehicle (*n* = 8) and Tyr-Pro (*n* = 10) are depicted as closed circle, square and triangle, respectively. Two data points corresponding to mice out of normal distribution in the control group [shown as open square in (**d**)] were eliminated as per the interquartile outlier test. Details of the test are described in supplemental information. Data are shown as median (solid bar), and first and third quartiles (dotted lines). Statistical significance was determined by Fisher’s PLSD test and Mann–Whitney U test for Y-maze test and passive avoidance test, respectively. ***p < 0.001 vs. sham control and ^#^*p* < 0.05 vs. Aβ_25-35_ alone. *N.S*. indicates no significance.

Acute Alzheimer’s disease (AD) has been typically simulated in mice by inducing cognitive impairment through Aβ_25-35_ injection (6 nmol per mouse *i.c.v*.)^7,8^, and soy dipeptide Tyr-Pro^1^ was orally administered at the dose of 100 mg/kg twice a day, as indicated in Fig. 1a. Regarding the Y-maze test, Tyr-Pro significantly improved reduced spontaneous alternation induced by Aβ (Fig. 1c) (*p* < 0.05), while the administration for 16 days did not affect locomotive activity of mice (no significant differences were noted in the number of total entries in the long arm between groups, Fig. 1b). This indicated that BBB transportable soy dipeptide Tyr-Pro can be potentially active in preventing reduction of working memory in mice.

Next, the step-through type passive avoidance test^9^ was performed to evaluate the benefits of Tyr-Pro in long-term memory in Aβ_25-35_-injected AD model mice. The interquartile outlier test allowed the elimination of two mice that showed unusually higher acquisition trial latency (Fig. 1d), and the three groups (sham, vehicle and Tyr-Pro groups) displayed no significant differences in the spectrum of step-through latency in the acquisition trial on day 15. On the 16th day, a significant impairment in the long-term memory or shortened latency time (*p* < 0.001) compared to the sham group was observed in Aβ_25-35_-injected mice (Fig. 1e). In contrast, the step-through latency time in Aβ_25-35_-injected mice did not significantly reduce upon Tyr-Pro administration (*p* < 0.05) (Fig. 1e). This indicated that Tyr-Pro could serve as a player in preventing long-term memory impairment or Aβ_25-35_-induced AD cognitive deficiency in mice.

Thus far, few peptides have been reported that exhibit a protective effect against cognitive decline. Mizushige et al.^10^ reported anxiolytic-like activity of Tyr-Leu in ddY mice via activation of serotonin 5-HT_1A_, dopamine D_1_ and γ-aminobutanoic acid (GABA) receptors. Effects of Leu-His^2^ on microglial activation and emotional disturbance and preventive effects of Met-Lys-Pro^3^ and Trp-Tyr^4^ on cognitive decline allowed us to speculate the potential effect of small peptides on brain health; however, no substantial evidence exists for their intact BBB transport and presence (or distribution) in brain parenchyma of orally administered peptides. Our previous study was the first evidential report that dipeptides possessing Pro (Gly-Pro and Tyr-Pro) from 18 dipeptide candidates can cross the BBB system in intact form, followed by the distribution in the hippocampus and cerebral cortex in peptide-perfused mice brain^1^. Moreover, in our preliminary data, Tyr-Pro was absorbed into blood circulation in its intact form in ICR mice after its single oral administration (shown in Supplemental Fig. 1), which suggesting a possible access of orally administered Tyr-Pro to brain tissue. Hence, the present study clearly demonstrated, for the first time, that BBB-transportable and orally absorbed Tyr-Pro can improve cognitive impairment in Aβ_25-35_-induced mice.

**Fig. 2:**
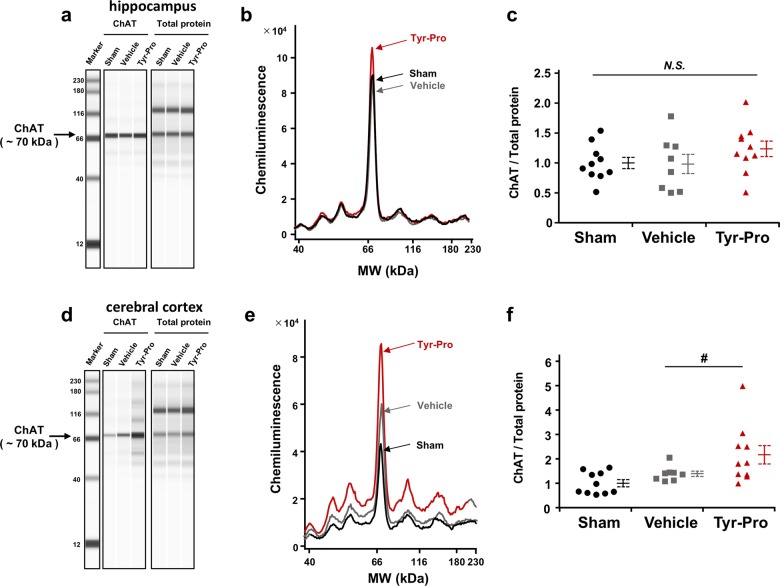
Effect of oral administration of Tyr-Pro (100 mg/kg, p.o.) for 16 days on the protein levels of choline acetyltransferase (ChAT) in Aβ_25-35_-induced model mice. Protein levels of ChAT in the hippocampus (**a**–**c**) and the cerebral cortex (**d**–**f**) were measured using a Wes instrument based on capillary electrophoresis immunoassay as described in supplemental information. The chemiluminescent signal is displayed as a virtual blot-like image (**a**, **d**) and eletropherogram (**b**, **e**) based on the molecular weight. Protein expression of ChAT was normalised by the electropherogram peak area of applied total protein in each lane, and the data are expressed as the ratio against the sham group. All data are presented as plot with the mean (solid bar) ± standard error (dotted lines). Statistical significance determined by Fisher’s PLSD test is represented as ^#^*p* < 0.05 vs. Aβ_25-35_ alone. *N.S*. indicates no significance.

Considering that AD therapeutic drugs, such as donepezil^11^ targeted acetylcholinesterase (AChE) to increase acetylcholine, neurotransmitter metabolism, such as acetylcholine metabolism in the central nerve system (CNS) at memory controlling regions in brain^6^ might be a possible target of Tyr-Pro. However, it was impossible to directly monitor acetylcholine in the present study, because acetylcholine is instable and quickly metabolized to choline and acetate by AChE, and a tactical sampling techniques, such as microdialysis in living mice^12^, is essential. Thus, the effect of Tyr-Pro on protein expression of choline acetyltransferase (ChAT) and AChE in the hippocampus and cerebral cortex of mouse brain was evaluated using a micro-capillary protein electrophoresis system in this study. As shown in Fig. 2d–f, the expression of ChAT in the cerebral cortex in Tyr-Pro group was significantly higher than that in vehicle group (*p* < 0.05), with an increasing tendency of ChAT protein accumulation in the hippocampus in the Tyr-Pro group (*p* = 0.116) (Fig. 2a–c). In contrast, no significant changes in AChE expression were observed in both regions of the brain (Supplemental Fig. 2). Collectively, we speculate that long-term (16 days) administration of BBB-transportable Tyr-Pro in the acute AD model mice may ameliorate Aβ-impaired acetylcholine metabolism by promoting acetylcholine production, particularly in the cerebral cortex, thereby improving both short- and long-term memory impairment in the CNS.

In conclusion, the present study demonstrates for the first time that soy dipeptide, Tyr-Pro, can potentially improve impaired cognitive deficits in both working and long-term memories in Aβ-injected AD model mice. Although the present study provides evidence of in vivo brain benefits of Tyr-Pro, possibly through cholinergic neurotransmission pathway via enhanced ChAT protein expression, acetylcholine amount in brain tissue, the involvement of other possible pathways such as glucose^13^ and *N*-methyl-d-aspartate metabolisms^14^, and influence of composed amino acids should be evaluated both in vivo and in vitro when probing for Tyr-Pro-induced brain health as its intact peptidic form. In addition, further investigations into the bioavailability (absorption into blood and accumulation in brain) of Tyr-Pro after long-term administration and the mechanism of Tyr-Pro on the acetylcholine system in vitro experiments are needed. Taken together, possible memory-benefits of Tyr-Pro should be considered when examining the physiological functions of dietary small peptides.

## Methods

All the animal procedures were performed in accordance with the National Institutes of Health guidelines for the use of experimental animals. The experimental protocol was reviewed and approved by the Animal Studies Committee of Nihon Bioresearch Inc. (Study No. 390066, Gifu, Japan). Five-week-old male ddY mice with 23–28 g body weight (Japan SLC Inc., Shizuoka, Japan) were used in this study. The experimental schedules are shown as Fig. 1a. Tyr-Pro (100 mg/kg) was orally administered twice a day for 16 days, except for days of *i.c.v*. injection of Aβ_25–35_ peptide and behavioural tests (Tyr-Pro administration once a day). The mice received *i.c.v*. injection of Aβ_25–35_ peptide at 6 nmol/mouse on the 7th day and Tyr-Pro administration was performed after recovery from anaesthesia, according to a previous report with several modifications^15^. Spontaneous alternation performance (Y-maze test) was started at 60 min after Tyr-Pro administration on the 14th day. Passive avoidance test (acquisition trial on the 15th day and retention trial on the 16th day) was started at 60 min after Tyr-Pro administration on each day. After the passive avoidance test, hippocampus and cerebral cortex of mouse brain were taken and stored at −80 °C until analysis. Other detail methods are available in supplemental information.

## Supplementary information

Supplemental information

## Data Availability

The data supporting the findings reported herein are available on reasonable request from the corresponding author.
