# Supplementary material for: Brain-transportable soy dipeptide, Tyr-Pro, attenuates amyloid β peptide25-35-induced memory impairment in mice
Source: NPJ Sci Food. 2020 May 1;4:7. doi: 10.1038/s41538-020-0067-3 (PMC7195433; doi:10.1038/s41538-020-0067-3)
Supplement: Supplementary file 1 — Supplemental information [file 41538_2020_67_MOESM1_ESM.pdf]

Supplemental information

Brain-transportable soy dipeptide, Tyr-Pro, attenuates amyloid β peptide$_{25-35}$-induced memory impairment in mice

Authors: Mitsuru Tanaka[1], Hayato Kiyohara[1], Atsuko Yoshino[1], Akihiro Nakano[1], Fuyuko Takata[2], Shinya Dohgu[2], Yasufumi Kataoka[2], Toshiro Matsui[1]*

Affiliations:

[1]Laboratory of Food Analysis, Department of Bioscience and Biotechnology, Faculty of Agriculture, Graduate School of Kyushu University, 744 Motooka, Nishi-ku, Fukuoka 819-0395, Japan

[2]Department of Pharmaceutical Care and Health Sciences, Faculty of Pharmaceutical Sciences, Fukuoka University, 8-19-1 Nanakuma, Jonan-ku, Fukuoka 814-0180, Japan

[*]**Correspondence**: Toshiro Matsui, PhD, Laboratory of Food Analysis, Department of Bioscience and Biotechnology, Faculty of Agriculture, Graduate School of Kyushu University, 744 Motooka, Nishi-ku, Fukuoka 819-0395, Japan

E-mail: tmatsui@agr.kyushu-u.ac.jp

Fax: +81-92-802-4752

Methods

All procedures were performed in accordance with the National Institutes of Health guidelines for the use of experimental animals. The experimental protocol was reviewed and approved by the Animal Studies Committee of Nihon Bioresearch Inc. (Study No. 390066).

Animals

Five-week-old male ddY mice with 23–28 g body weight (Japan SLC Inc., Shizuoka, Japan) were used in this study. All mice were housed for 1 week under controlled temperature at 20.0–26.0°C, humidity at 40.0–70.0%, and light schedule for a 12 h period from 6:00 am to 6:00 pm. The mice were fed MF diet (Oriental Yeast Co., Ltd., Tokyo, Japan) and water *ad libitum*.

Experimental schedules

Experimental schedules are depicted in Figure 1a. Tyr-Pro (100 mg/kg) was orally administered twice a day for 16 days, except for days of *i.c.v.* injection of $A\beta_{25\text{–}35}$ peptide and the behavioural tests (Tyr-Pro administration once a day). The mice received *i.c.v.* injection of $A\beta_{25\text{–}35}$ peptide at 6 nmol/mouse on the 7th day and Tyr-Pro administration was performed after recovery from anesthesia. Spontaneous alternation performance (Y-maze test) was started at 60 min after Tyr-Pro

administration on the 14th day. Passive avoidance test (acquisition trial on the 15th day and retention trial on the 16th day) was started at 60 min after Tyr-Pro administration on each day. After the passive avoidance test, all the mice were sacrificed, and brain tissues were removed and stored -80ºC until further analysis. Sham control mice were treated with distilled water, and *i.c.v.* injection of distilled water. Vehicle control mice received distilled water and an *i.c.v.* injection of $A\beta_{25–35}$ peptide.

$A\beta_{25–35}$ peptide induced memory impairment

Aβ-protein (Human 25–35) (Peptide Institute, Osaka, Japan) was dissolved in distilled water (at the final concentration of 2 mM) and incubated at 37°C for 96 h. *i.c.v.* injection was performed as described previously with several modifications[11]. Briefly, each mouse was anesthetized by intraperitoneal injection of nembutal in saline and subcutaneous injection of levobupivacaine, and placed in a stereotaxic frame (Narishige Inc., Tokyo, Japan). Needle (28 gauge) was injected into following position: 1 mm right of the midline, 0.2 mm posterior, and 2.5 mm depth from bregma. $A\beta_{25–35}$ solution (3 μL, 6 nmol/mouse) was then injected *i.c.v.* at a rate of 1 μL/min using a syringe pump. The needle was kept in place for additional 3 min and then withdrawn.

Spontaneous alternation behaviour

To evaluate the short-term memory of mice, a Y-maze test was performed on the 7th day after *i.c.v.* injection of Aβ. The maze consisted of polyvinyl plastic and had three arms (395 mm deep, 120 mm high, 45 mm wide at the bottom, and 100 mm wide at the top) at angles of 120°. One hour after administration of Tyr-Pro or distilled water, mice were placed at the end of one arm and allowed to move freely for 8 min. The sequence of arm entry was counted manually to calculate the total number of entries and the alternation ratio (ratio of actual alternations to maximum alternation, i.e., total number of entries -2).

Step-through type passive avoidance test

One day after the Y-maze test, long-term memory of the mice was assessed by a passive avoidance test. The apparatus consisted of one illuminated (100 mm wide, 100 mm deep, and 300 mm high) and one dark (240 mm wide, 245 mm deep, and 300 mm high) chamber with grid floors, which were separated by a guillotine door. During the acquisition trial, each mouse was placed in the illuminated chamber, the guillotine door was opened after 10 s and the initial latency to enter the dark compartment was recorded. When the mouse moved completely into the dark compartment, the door was closed. Then, the mouse received an electric shock (0.2 mA, 2 s duration, scrambled) and was then returned to its cage. The test trial was repeated 24 h later by placing the mouse in the

illuminated chamber and measuring the latency period to enter the dark compartment up to 300 s.

Quantification of ChAT and AChE protein expression by WES system

Protein levels of ChAT and AChE in brain tissue were quantitated with a Wes instrument based on a capillary electrophoresis immunoassay (ProteinSimple Co., San Jose, CA, USA). Hippocampal and cerebral cortical brain tissues were homogenized in 1.2 mL RIPA buffer [150 mM NaCl, 50 mM Tris (pH 7.4), 1.0% NP-40, 0.5% sodium deoxycholate, and 0.1% SDS] containing 1 mM phenylmethylsulfonyl fluoride (Nacalai Tesque Co. Kyoto, Japan) and 1% protease inhibitor cocktail (Nacalai Tesque Co.) with a Polytron homogenizer (KINEMATICA AG, Luzern, Switzerland) with 20,000 rpm for 90 s × 2 times at 4°C. After centrifugation of the homogenate at 14,000 × *g* for 20 min at 4°C, the supernatants of cerebral cortex and hippocampus were diluted to 0.5 and 1.0 μg/μL of protein with 0.1× Sample Diluent buffer (ProteinSimple, Co.), respectively. Four volumes of diluted samples were mixed with 1 volume of a 5× Fluorescent Master Mix containing 5% SDS and 200 mM dithiothreitol, followed by the denaturation at 95°C for 5 min. The Wes measurement was operated with a 12–230 kDa Separation Module (8 × 25 mm capillary cartridge, ProteinSimple Co.), according to the manufacturer's instructions with several modifications. Wes reagents (biotinylated

ladder and primary antibodies) were dispensed in a microplate and subjected to a Wes automated capillary electrophoresis, followed by an automated immune-detection using a horseradish peroxidase-conjugated anti-rabbit secondary antibody and chemiluminescent substrate. Primary antibodies for ChAT (1:50, ab50412, Abcam, Cambridge, UK) and AChE (1:50 dilution, ab183591, Abcam) were used. For total protein detection, pentafluorophenyl ester-biotin labeling reagent that can attach to all the applied proteins was used. At the end of the run, chemiluminescent signal was displayed as virtual blot-like image and eletropherogram based on the molecular weight using a ProteinSimple Compass software (ProteinSimple Co.). Protein expression was normalised by electropherogram peak area of applied total protein in each lane, and the data are expressed as the ratio against sham group. All data used in this study are expressed in Supplemental Figure 3 as uncropped virtual blot-like images.

Statistical analysis

Behavioural data are presented as plot with median, and first and third quartiles. Data for protein levels are presented as plot with the mean ± standard error. A one-way analysis of variance (ANOVA) was performed to analyse the statistical difference between groups, followed by Fisher's PLSD test for Y-maze test. Regarding passive avoidance test, Mann-Whitney U test was used.

Because two mice in control group expressed unusual behaviour in both acquisition and retention trials of passive avoidance test at 15–16th day, and the corresponding data points were out of normal distribution in control group (shown as open square in Figure 2d) they were eliminated as per the interquartile outlier test. A *p*-value of < 0.05 was considered statistically significant.

Supplemental Figure 1

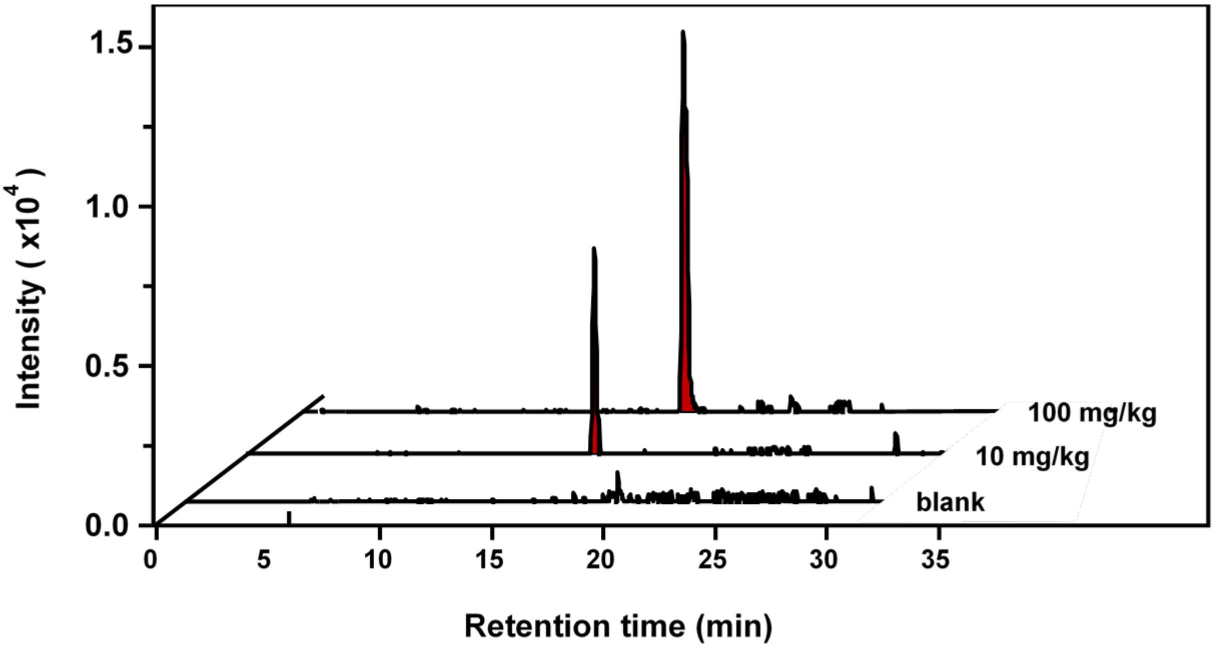

Supplemental Figure 1 Stacked intensity-time chromatograms of Tyr-Pro in plasma at 15 min after oral administration (blank, 10 and 100 mg/kg B.W.).

Plasma was corrected at 15 min after a single oral administration at each dose of 10 or 100 mg/kg B.W. in 7 wk-old ICR mice (16 h starvation before the experiment). The plasma was subjected to an ultrafiltration using an Amicon Ultra 0.5-mL-3K centrifugal filter followed by a liquid chromatograph-time-of-flight-mass spectrometry assay (Tanaka, M. et al. ***Mol. Nutr. Food. Res, 59,*** 1541-1549).

Supplemental Figure 2

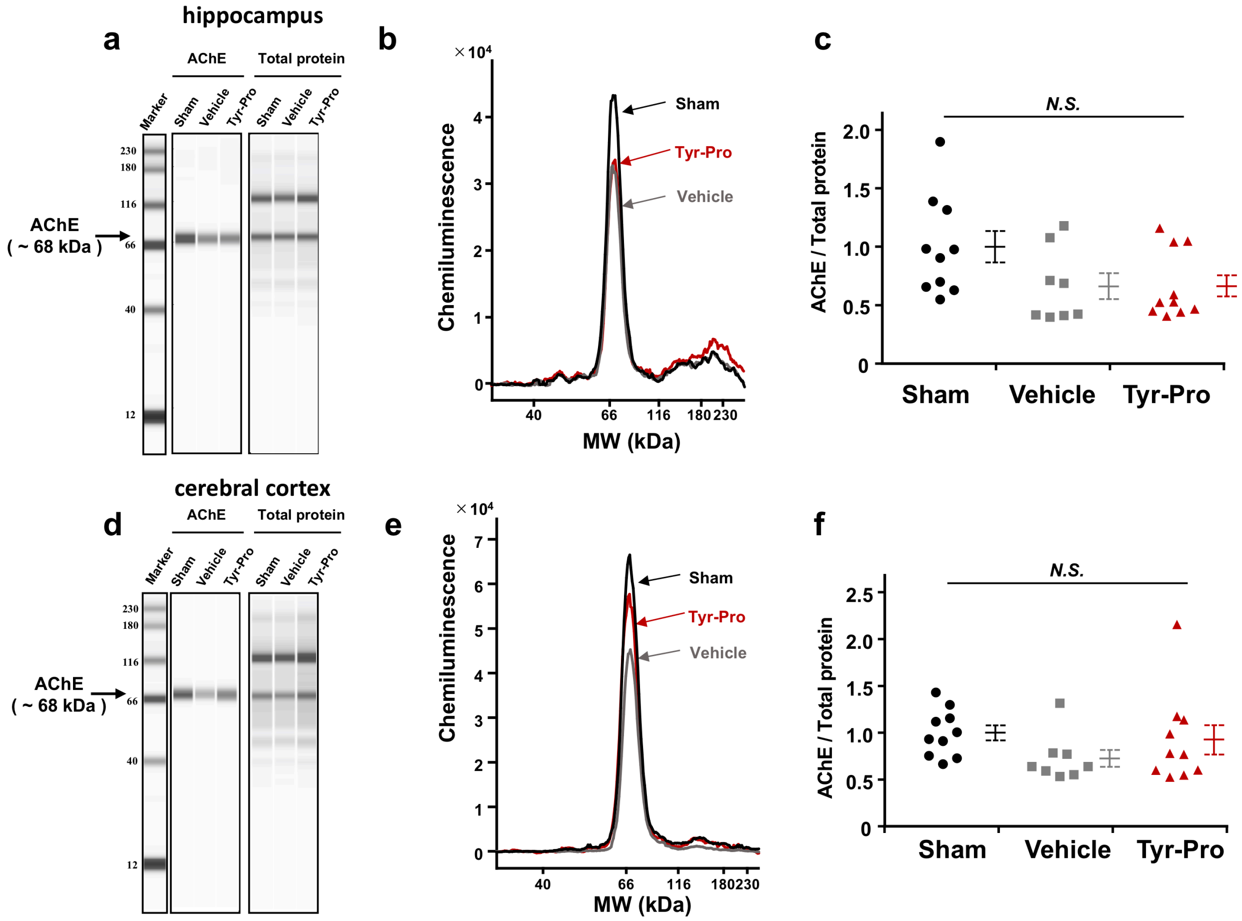

Supplemental Figure 2 Effect of oral administration of Tyr-Pro (100 mg/kg, *p.o.*) for 16 days on the protein expression of acetylcholinesterase (AChE) in A$\beta_{25\text{-}35}$-induced model mice.

Protein levels of AChE in the hippocampus (a–c) and the cerebral cortex (d–f) was measured with a Wes instrument based on a capillary electrophoresis immunoassay, as described in supplemental information. The chemiluminescent signal is displayed as a virtual blot-like image (a, d) and eletropherogram (b, e) based on the molecular weight. The protein expression of AChE was normalised by electropherogram peak area of applied total protein in each lane, and the data are

expressed as the ratio against sham group. All data are presented as plot with the mean (solid bar) ± standard error (dotted lines). Statistical differences between groups were analysed by Fisher's PLSD test. *N.S.* indicates no significance.

Supplemental Figure 3

Hippocampus

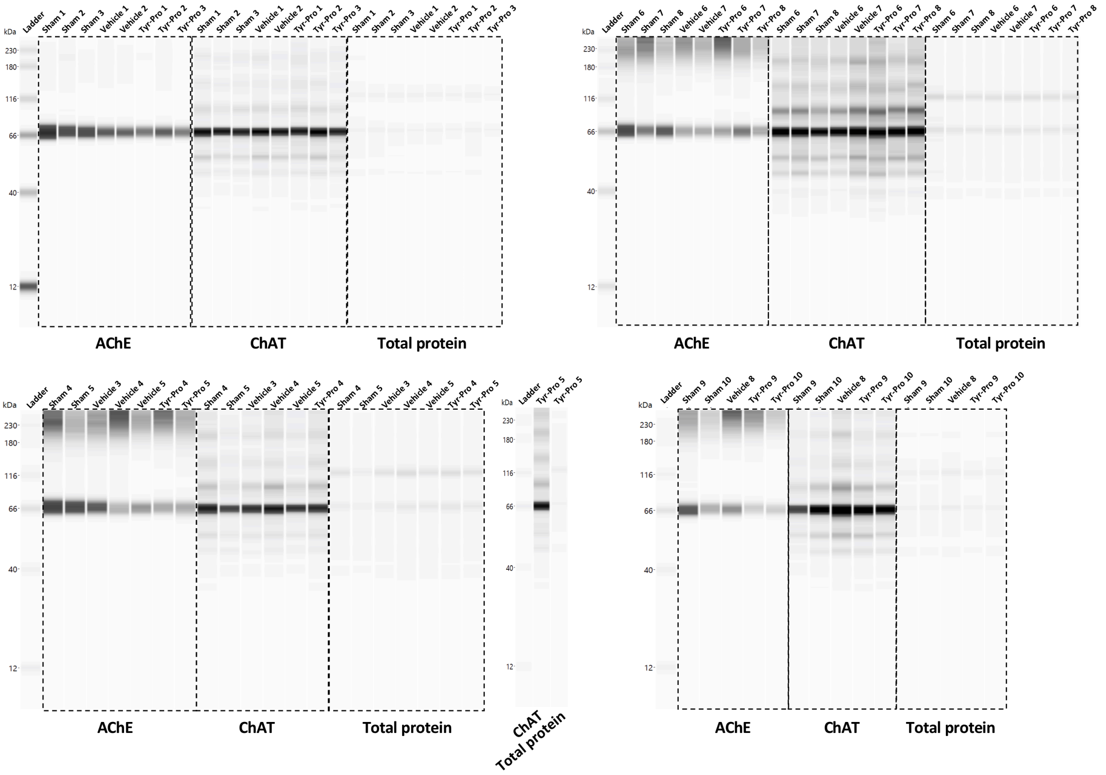

Cortex

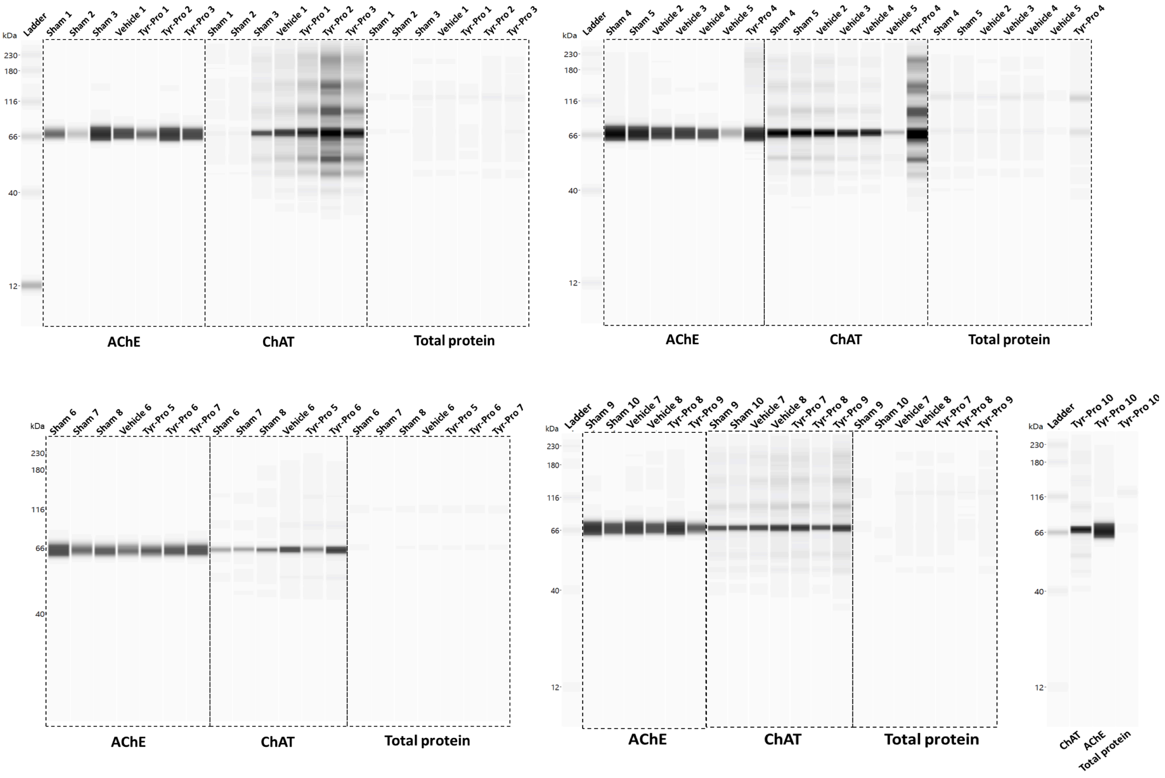

Supplemental Figure 3 Uncropped virtual blot-like images for protein expression of choline acetyltransferase (ChAT) and acetylcholinesterase (AChE) by a Wes instrument.

Protein expression of ChAT, AChE, and total protein in the hippocampus and the cerebral cortex were evaluated by a Wes instrument based on capillary electrophoresis immunoassay as described in the Supplemental information. All the uncropped virtual blot-like images used in Figure 2 and Supplemental Figure 2 are provided.
